# *Toxoplasma gondii* Tetravalent Chimeric Proteins as Novel Antigens for Detection of Specific Immunoglobulin G in Sera of Small Ruminants

**DOI:** 10.3390/ani9121146

**Published:** 2019-12-13

**Authors:** Lucyna Holec-Gąsior, Bartłomiej Ferra, Weronika Grąźlewska

**Affiliations:** Department of Molecular Biotechnology and Microbiology, Faculty of Chemistry, Gdańsk University of Technology, Narutowicza 11/12 Str., 80–233 Gdańsk, Poland; barferra@pg.edu.pl (B.F.); wergrazl@student.pg.edu.pl (W.G.)

**Keywords:** chimeric proteins, ELISA, goat, sheep, *Toxoplasma gondii*, toxoplasmosis

## Abstract

**Simple Summary:**

*Toxoplasma gondii* infection leads to large economic losses in the sheep and goat industry worldwide and is considered to be one of the main causes of infectious ovine and caprine abortion. Moreover, in countries where sheep and goat meat are frequently consumed, *T. gondii* infection in small ruminants may also pose a public health risk. Due to its medical and veterinary importance, it is essential to study the seroprevalence of *T. gondii* infection among farm animals and humans. This requires the development of new, low-cost diagnostic methods such as enzyme immunoassays based on recombination antigens. Thus, the study aimed to evaluate the reactivity of four different tetravalent chimeric proteins containing immunodominant regions from the AMA1 (apical membrane antigen 1), SAG2 (surface antigen 2), GRA1 (dense granule antigen 1), GRA2 (dense granule antigen 2), and ROP1 (rhoptry antigen 1) *T. gondii* antigens with specific IgG from the sera of small ruminants. The results demonstrate that an IgG ELISA (enzyme-linked immunosorbent assay) based on one of these chimeric proteins (AMA1-SAG2-GRA1-ROP1) may be a useful test for the determination of *T. gondii* infection in small ruminants.

**Abstract:**

The detection of *Toxoplasma gondii* infection in small ruminants has important significance for public health and veterinary medicine. This study, for the first time, describes the reactivity of four tetravalent chimeric proteins (AMA1_N_-SAG2-GRA1-ROP1, AMA1_C_-SAG2-GRA1-ROP1, AMA1-SAG2-GRA1-ROP1, and SAG2-GRA1-ROP1-GRA2) containing immunodominant regions from the AMA1 (apical membrane antigen 1), SAG2 (surface antigen 2), GRA1 (dense granule antigen 1), GRA2 (dense granule antigen 2), and ROP1 (rhoptry antigen 1) with specific IgG antibodies from the sera of small ruminants with the use of an indirect enzyme-linked immunosorbent assay (ELISA). The reactivity of individual chimeric antigens was analyzed in relation to the results obtained in IgG ELISA based on a *Toxoplasma* lysate antigen (TLA). All chimeric proteins were characterized by high specificity (between 96.39% to 100%), whereas the sensitivity of the IgG ELISAs was variable (between 78.49% and 96.77%). The highest sensitivity was observed in the IgG ELISA test based on the AMA1-SAG2-GRA1-ROP1. These data demonstrate that this chimeric protein can be a promising serodiagnostic tool for *T. gondii* infection in small ruminants.

## 1. Introduction

*Toxoplasma gondii* infection is widely prevalent in humans and warm-blooded animals worldwide [1]. In most cases, this infection does not cause severe illness. However, serious clinical symptoms can result when a primary infection occurs during pregnancy or when the host immune response is compromised [1]. Infection by *T. gondii* is relatively common in small ruminants [2,3], causing reproductive problems and economic losses in sheep and goat herds [4]. Primary *T. gondii* infections in livestock, in particular sheep and goats, pose a health risk to these animals, as the infection is known to cause abortions, stillbirths, and neonatal mortalities [2]. In the United Kingdom, for example, ovine toxoplasmosis causes up to 2% of fetal loss per annum [4,5]. Furthermore, *T. gondii* infection can affect humans primarily via the consumption of animal products from certain species, including small ruminants. Pork, mutton, and goat meat containing tissue cysts of the parasite is considered to be the main source of *T. gondii* infection in humans in Europe and the United States [1]. Therefore, regular monitoring of the infection in these animal populations is advisable to control human and animal toxoplasmosis. This monitoring can be provided with the use of new and more sensitive tools (e.g., recombinant antigens of the parasite) for the detection of specific immunoglobulins in sera of different individuals. Currently, most of the commercially available serological kits for human and animal diagnosis of *T. gondii* infection utilize *Toxoplasma* lysate antigens (TLAs) isolated from tachyzoites obtained from the peritoneal fluid of an infected mouse or from in vitro cell cultures. Although the TLA is characterized by high sensitivity and specificity in an enzyme-linked immunosorbent assay (ELISA), its disadvantages are the high cost and lengthy production time, as well as the need to maintain parasite cultures. Thus, recombinant proteins of *T. gondii* could be an alternative source of antigens [6]. This should prove highly beneficial to improving the standardization of the method as the antigen composition of the test will be precisely known. Moreover, the production cost of antigens would be reduced. Chimeric proteins containing different immunoreactive epitopes from various antigens of the parasite are a new generation of recombinant antigens with diagnostic potential. These preparations are obtained as a result of combining two or more fragments (usually of various antigens) into the so-called fusion gene. As a result of the expression of such a fusion gene, a chimeric protein is produced, which should be recognized by antibodies against individual antigens. This should result, among other things, in increasing the sensitivity of immunoassays based on chimeric proteins. Furthermore, the combination of epitopes or immunodominant regions of different antigens characteristic of various stages of the *T. gondii* life cycle is an optimal strategy to overcome the antigen complexity of the parasite. Over the past 10 years, several different chimeric recombinant proteins have been used for the detection of *T. gondii*-specific antibodies in humans [6,7,8,9,10,11] and animal sera [12]. Nonetheless, the use of chimeric proteins in the diagnosis of *T. gondii* infection in farm animals is a relatively new approach. Therefore, in this study, we evaluated the diagnostic usefulness of four tetravalent recombinant chimeric proteins (AMA1_N_-SAG2-GRA1-ROP1, AMA1_C_-SAG2-GRA1-ROP1, AMA1-SAG2-GRA1-ROP1, and SAG2-GRA1-ROP1-GRA2) composed of a combination of different fragments from five well-characterized antigens, including different regions of apical membrane antigen (AMA1), surface antigen (SAG2), rhoptry antigen (ROP1), and two dense granule antigens (GRA1 and GRA2) (Table 1). The selection of these antigens for the construction of chimeric proteins was based on earlier immunoassay results [13,14,15,16]. The potential of the above-mentioned *T. gondii* recombinant chimeric proteins for the detection of specific antibodies in sera of small ruminants was evaluated by the IgG ELISA test. 

## 2. Materials and Methods

### 2.1. Toxoplasma gondii Chimeric Recombinant Proteins and Toxoplasma Lysate Antigen

All tetravalent chimeric recombinant proteins (AMA1_N_-SAG2-GRA1-ROP1, AMA1_C_-SAG2-GRA1-ROP1, AMA1-SAG2-GRA1-ROP1, and SAG2-GRA1-ROP1-GRA2), containing a cluster of six histidine residues for the purification by metal-affinity chromatography at N- and C-termini, were obtained using the methods described previously [9]. The chimeric proteins were analyzed by SDS-PAGE on 10% acrylamide gels and stained with Coomassie blue. The concentration of purified recombinant proteins was determined using Bradford reagent (Bio-Rad) according to the manufacturer’s recommendation.

*Toxoplasma* lysate antigen (TLA) from tachyzoites (strain RH), serving as a source of the parasite’s antigens, was prepared according to the methods described by Dziadek et al. [17]. Briefly, the tachyzoites were repeatedly frozen and thawed to lyse the parasite cells. After the centrifugation of resulting cell lysates (20 min at 10,000 g and 4 °C) the supernatant containing native *T. gondii* antigens was stored at −80 °C and next used in ELISA evaluations. The concentration of proteins in the TLA preparation was determined using Bradford reagent (Bio-Rad) according to the manufacturer’s recommendation. 

### 2.2. Serums Samples of Small Ruminants

A total of 176 serum samples of small ruminants (90 from sheep and 86 from goat) were received from the Veterinary Hygiene Station (Gdańsk, Poland). These sera were obtained from epidemiological studies conducted on a livestock population from the northern region of Poland [18]. All sera were classified on the levels of specific IgG antibodies according to the method described previously (an in-house ELISA assay [18]) and to the manufacturer’s description of the diagnostic commercial kits: an agglutination test (Toxo-Screen DA, bioMérieux, Marcy-l’Étoile, France) and an immunofluorescence test using slides coated with *T. gondii* antigen (Toxo-Spot IF, bioMérieux, Marcy-l’Étoile, France). Based on the results of the diagnostic tests, the sera were divided into seropositive and seronegative groups as follows: group I_S_—48 sera from naturally infected sheep (IgG positive), group I_G_—45 sera from naturally infected goat (IgG positive), group II_S_—42 sera from healthy sheep (IgG negative) and group II_G_—41 sera from healthy goat (IgG negative). Moreover, all serum samples were also seronegative for anti-*Neospora caninum* specific antibodies, as was determined by using a commercial competitive-inhibition enzyme-linked immunosorbent assay (*Neospora caninum* Antibody Test Kit, cELISA, VMRD, Inc, Pullman, WA, USA). Furthermore, an additional fifteen ovine sera (seronegative for *T. gondii* and seropositive for *N. caninum*) were used for estimating the chimeric recombinant proteins cross-reactivity with specific *Neospora caninum* antibodies.

### 2.3. IgG ELISA

An in-house immunoglobulin G (IgG) ELISA was used to determine the reactivity of chimeric proteins, which was carried out as previously described [12]. Each recombinant chimeric protein was used at a concentration of 2.5 μg/ml and their reactivity was compared to the reactivity of TLA used at a concentration of 1 μg/ml. Immune complexes were identified using secondary anti-sheep and anti-goat peroxidase-labeled conjugates (JacksonImmuno Research, Newmarket, Suffolk, UK) diluted 1:32,000 and 1:16,000, respectively.

Each serum sample was tested twice, and the results were determined by calculating the mean value of the optical density (OD) at 492 nm for duplicate wells. Moreover, reference sera (positive and negative) on each ELISA plate were used as controls in all experiments. 

### 2.4. Statistical Analysis

Statistical analysis was performed using SigmaPlot 14.0 software (Systat Software). Means and ranges of the absorbance measurements for the sera used in the individual studies are given. Receiver operating characteristic (ROC) curve analysis was performed to obtain the area under the curve (AUC), the sensitivity and the specificity of all the IgG ELISAs based on the four different chimeric proteins and TLA preparations.

## 3. Results

A total of 176 serum samples from small ruminants were examined. The four recombinant chimeric proteins (AMA1_N_-SAG2-GRA1-ROP1, AMA1_C_-SAG2-GRA1-ROP1, AMA1-SAG2-GRA1-ROP1, and SAG2-GRA1-ROP1-GRA2) and the TLA reacted with anti-*T. gondii* antibodies from sheep and goat sera with different sensitivity and specificity (Table 2). The above-mentioned recombinant antigens and TLA preparation reacted with specific IgG with different sensitivity and specificity (Table 2). The 100% specificity calculated for all tested seronegative sera was observed only in the IgG ELISAs using the AMA1-SAG2-GRA1-ROP1 chimeric protein and the TLA. Moreover, the same high specificity was noted for the immunoassay based on AMA1_N_-SAG2-GRA1-ROP1 when testing goat sera. Whereas, for all the tested seropositive sera of small ruminants, the highest sensitivity (96.77%) was observed in the IgG ELISA test based on the AMA1-SAG2-GRA1-ROP1 chimeric protein. This result was comparable to the sensitivity result of the TLA-based IgG ELISA (97.85%). Furthermore, relatively high reactivity was noticed for two of the remaining chimeric proteins containing different fragments of the AMA1 antigen, AMA1_N_-SAG2-GRA1-ROP1, and AMA1_C_-SAG2-GRA1-ROP1, at 93.55% and 95.70%, respectively. Lower sensitivity, at a level of 78.49% was observed for the IgG ELISA based on the SAG2-GRA1-ROP1-GRA2 recombinant protein. This chimeric protein reacted with high sensitivity with the anti-*T. gondii* antibodies from sheep sera (97.92%), whereas the specific IgG antibodies were detected only in the case of 26 goat sera out of the 45 tested, resulting in 57.78% sensitivity of this IgG ELISA. 

Furthermore, to evaluate the possibility of the cross-reactivity of four recombinant chimeric proteins with specific antibodies against *N. caninum*, species of parasite closely related to the *T. gondii*, 15 additional sheep serum samples containing specific anti-*N. caninum* IgG were tested in ELISA. None of these sera were found to score above the cutoff (range absorbance: 0.125–0.229, mean absorbance 0.189, 0.145, 0.208, 0.215 for AMA1_N_-SAG2-GRA1-ROP1, AMA1_C_-SAG2-GRA1-ROP1, AMA1-SAG2-GRA1-ROP1, and SAG2-GRA1-ROP1-GRA2, respectively).

## 4. Discussion

Serological tests are one of the basic diagnostic methods currently used to detect *T. gondii* infection in humans and various animal species. Commercially available serological kits for recognition of specific antibodies mainly use the whole extracts of tachyzoites. Various forms of recombinant antigens, such as chimeric proteins are very promising tools that can be used in diagnostic tests instead of native antigens of the parasite. Moreover, the commercially available diagnostic tests due to its price are not generally applicable in the serodiagnosis of *T. gondii* infection in animals, which requires the analysis of a large number of samples. For this reason, many research groups are currently working on new diagnostic tools, which are mainly recombinant antigens. Compared to the native antigens their production is much easier, cheaper, faster, and safer. An additional advantage of the recombinant antigens is an easier way to standardize assays as well as the possibility of protein selection characteristics for the development form of the parasite.

The study aimed to estimate the capability of four tetravalent chimeric proteins (AMA1_N_-SAG2-GRA1-ROP1, AMA1_C_-SAG2-GRA1-ROP1, AMA1-SAG2-GRA1-ROP1, and SAG2-GRA1-ROP1-GRA2) of *T. gondii* to detect specific IgG from sera of small ruminants in ELISA assays. In our previous paper, we have shown the usefulness of these recombinant antigens for serodiagnosis of *T. gondii* infection in humans [9]. Because of the very promising results of our study, we decided to evaluate also their reactivity with specific IgG from ovine and caprine sera. Three antigens (SAG2 (P22), GRA1, and ROP1) used for the construction of above mentioned tetravalent chimeric proteins were previously tested, as single preparations and a mixture of these antigens, in an IgG ELISA for detection of specific antibodies in naturally infected sheep [16]. The results showed that the cocktail of these three recombinant proteins (SAG2 + GRA1 + ROP1) could replace the TLA in serological tests for the diagnosis of *T. gondii* infection in sheep. To date, only our previous study showing the possibility of using recombinant chimeric proteins to detect *T. gondii* infection in farm animals can be found in the literature [12]. In that paper, we evaluated the diagnostic utility of five recombinant trivalent chimeric proteins (MIC1-MAG1-SAG1_S_, SAG1_L_-MIC1-MAG1, SAG2-GRA1-ROP1_S_, SAG2-GRA1-ROP1_L_, and GRA1-GRA2-GRA6) in the IgG ELISAs with sera from three different groups of livestock animals (horses, pigs, and sheep). Our result indicated that trivalent chimeric proteins were generally more reactive than mixtures of the same antigens. The most effective for the recognition of *T. gondii* infection was SAG2-GRA1-ROP1_L_, which could detect specific anti-*T. gondii* antibodies in 100%, 93.8%, and 100% of positive serum samples from horses, pigs, and sheep, respectively [12]. The next step in our research was the development of tetravalent chimeric proteins. Unfortunately, the results of the presented study showed that adding the fourth fragment of the dense granule antigen (GRA2) to the above mentioned trivalent chimeric protein did not improve its reactivity with specific antibodies from goat serum samples. However, when sheep sera were tested, the sensitivity of the IgG ELISA test based on SAG2-GRA1-ROP1-GRA2 chimeric protein was the same as the sensitivity of the TLA-based immunoassay. In this analysis, specific IgG antibodies were not detected in the case of only one ovine serum sample, resulting in a test sensitivity of 97.92%. Therefore, the results obtained in this study for the new tetravalent chimeric proteins confirm the results of our previous research on different trivalent recombinant antigens, which demonstrated that their reactivity with specific antibodies from animal sera was variable and mainly dependent on the animal species [12]. Moreover, these results demonstrate the difficulty with constructing one "universal" antigen that could be used to detect specific antibodies in the sera of various host species. One type of antigen that is well recognized by anti-*T. gondii* antibodies in one species of animals may be completely useless for the diagnosis of toxoplasmosis in another species. In this paper, very promising diagnostic results for the AMA1-SAG2-GRA1-ROP1 chimeric protein containing the largest fragment of the AMA1 (amino acid residues 68 to 569) were shown. The AMA1-SAG2-GRA1-ROP1 was better at detecting specific antibodies in the sera of small ruminants than two other chimeric antigens containing shorter fragments of AMA1 (AMA1_N_-SAG2-GRA1-ROP1, amino acids 68–287 and AMA1_C_-SAG2-GRA1-ROP1, amino acids 287–569). Therefore, the results of this work, as well as our previous studies, showed that in order to construct antigens with diagnostic utility a rational selection of protein fragments is of great importance [8,9,12]. Particular attention should be paid to the size of the antigen fragments, but also to their order in the final fusion construct, which was shown and discussed in our previous study [9]. Moreover, the AMA1-SAG2-GRA1-ROP1 chimeric protein seems to be the most promising and effective of all the published chimeric proteins of *T. gondii* having diagnostic utility. Our previous results showed that this protein can also be a good diagnostic tool for detecting IgG antibodies in human and mouse sera and possesses a high ability to detected IgM antibodies and determine the avidity index [9]. Thus, the AMA1-SAG2-GRA1-ROP1 has the potential to distinguish specific antibodies from sera of individuals during the early and chronic phases of *T. gondii* infection. Furthermore, no cross-reactions of this antigen with specific antibodies against *N. caninum* (the closely related parasite to the *T. gondii*) were found.

## 5. Conclusions

In summary, we conclude that the serum-based IgG ELISA using the AMA1-SAG2-GRA1-ROP1 tetravalent chimeric protein may be a useful test for the determination of *T. gondii* infection in small ruminants. However, further effort is needed before the IgG ELISA using the tetravalent chimeric protein will be commercially available as a tool for epidemiological studies.

## Figures and Tables

**Table 1:** Characteristic of the *T. gondii* tetravalent recombinant chimeric proteins.

Chimeric Proteins	Amino Acid Residues	Protein Characteristic
**AMA1_N_-SAG2-GRA1-ROP1**	68–287 AMA1 31–170 SAG2 26–190 GRA1 85–396 ROP1	899 aa Mw 97.95 kDa
**AMA1_C_-SAG2-GRA1-ROP1**	287–569 AMA1 31–170 SAG2 26–190 GRA1 85–396 ROP1	961 aa Mw 103.20 kDa
**AMA1-SAG2-GRA1-ROP1**	68–569 AMA1 31–170 SAG2 26–190 GRA1 85–396 ROP1	1182 aa Mw 128.42 kDa
**SAG2-GRA1-ROP1-GRA2**	31–170 SAG2 26–190 GRA1 185–396 ROP1 51–185 GRA2	806 aa Mw 86.32 kDa

aa—amino acids; Mw—molecular weight; AMA1—apical membrane antigen 1; SAG2—surface antigen 2; GRA1—dense granule antigen 1; GRA2—dense granule antigen 2; and ROP1—rhoptry antigen.

**Table 2:** The results of IgG ELISA (enzyme-linked immunosorbent assay) obtained for the *T. gondii* tetravalent recombinant chimeric proteins and the TLA (*Toxoplasma* lysate antigen) with the use of 176 serum samples of small ruminants (93 seropositive and 83 seronegative).

Results of IgG ELISA		Chimeric Recombinant Antigens and TLA				
		AMA1_N_-SAG2-GRA1-ROP1	AMA1_C_-SAG2-GRA1-ROP1	AMA1-SAG2-GRA1-ROP1	SAG2-GRA1-ROP1-GRA2	TLA
**Sheep Sera**	Group I_S_ = 48 ^A^	1.457 (0.175–2.700)	1.808 (0.081–2.308)	1.572 (0.150–2.611)	1.509 (0.108–2.897)	1.349 (0.237–2.256)
	Group II_S_ = 42 ^A^	0.222 (0.096–0.469)	0.132 (0.077–0.364)	0.212 (0.100–0.440)	0.232 (0.101–0.968)	0.269 (0.133–0.438)
	Cutoff	0.388	0.238	0.467	0.502	0.442
	Sensitivity	97.92%	95.83%	97.92%	97.92%	97.92%
	Specificity	97.62%	95.24%	100%	97.62%	100%
	AUC (ROC analysis)	0.9836	0.9678	0.9836	0.9777	0.9881
	PPV ^B^	97.92%	95.83%	100%	97.92%	100%
	NPV ^C^	97.62%	95.24%	97.67%	97.62%	97.67%
**Goat Sera**	Group I_G_ = 45 ^A^	0.991 (0.184–2.668)	1.201 (0.304–2.382)	1.142 (0.343–2.668)	1.160 (0.179–2.311)	1.046 (0.427–2.258)
	Group II_G_ = 41 ^A^	0.256 (0.127–0.795)	0.620 (0.304–1.128)	0.267 (0.127–0.535)	0.198 (0.109–0.353)	0.314 (0.196–0.464)
	Cutoff	0.546	0.336	0.534	0.533	0.489
	Sensitivity	88.89%	95.56%	95.56%	57.78%	97.78%
	Specificity	100%	97.56%	100%	95.12%	100%
	AUC (ROC analysis)	0.9981	0.9897	0.9900	0.8425	0.9989
	PPV ^B^	100%	89.58%	100%	92.86%	100%
	NPV ^C^	89.13%	95.24%	95.35%	67.24%	97.62%
**All Sera**	Sensitivity ^D^	93.55%	95.70%	96.77%	78.49%	97.85%
	Specificity ^E^	98.8%	97.59%	100%	96.39%	100%
	PPV ^B^	98.86%	97.80%	100%	96.05%	100%
	NPV ^C^	93.18%	95.29%	97.65%	80%	97.65%

^A^ Mean absorbance value (range); ^B^ positive predictive value PPV = true positive / (true positive + false positive); ^C^ negative predictive value NPV = true negative / (true negative + false negative); ^D^ calculated for all seropositive sera I_S_ + I_G_ = 93; ^E^ calculated for all seronegative sera II_S_ + II_G_ = 83.
